# Motivational Objects in Natural Scenes (MONS): A Database of >800 Objects

**DOI:** 10.3389/fpsyg.2017.01669

**Published:** 2017-09-26

**Authors:** Judith Schomaker, Elias M. Rau, Wolfgang Einhäuser, Bianca C. Wittmann

**Affiliations:** ^1^Department of Psychology, Justus Liebig University Giessen, Giessen, Germany; ^2^Institute of Physics, Chemnitz University of Technology, Chemnitz, Germany; ^3^Department of Neurophysics, Philipps University of Marburg, Marburg, Germany

**Keywords:** objects, scenes, motivational value, arousal

## Abstract

In daily life, we are surrounded by objects with pre-existing motivational associations. However, these are rarely controlled for in experiments with natural stimuli. Research on natural stimuli would therefore benefit from stimuli with well-defined motivational properties; in turn, such stimuli also open new paths in research on motivation. Here we introduce a database of Motivational Objects in Natural Scenes (MONS). The database consists of 107 scenes. Each scene contains 2 to 7 objects placed at approximately equal distance from the scene center. Each scene was photographed creating 3 versions, with one object (“critical object”) being replaced to vary the overall motivational value of the scene (appetitive, aversive, and neutral), while maintaining high visual similarity between the three versions. Ratings on motivation, valence, arousal and recognizability were obtained using internet-based questionnaires. Since the main objective was to provide stimuli of well-defined motivational value, three motivation scales were used: (1) Desire to own the object; (2) Approach/Avoid; (3) Desire to interact with the object. Three sets of ratings were obtained in independent sets of observers: for all 805 objects presented on a neutral background, for 321 critical objects presented in their scene context, and for the entire scenes. On the basis of the motivational ratings, objects were subdivided into aversive, neutral, and appetitive categories. The MONS database will provide a standardized basis for future studies on motivational value under realistic conditions.

## Introduction

When interacting with the world around us, a range of factors guide our behavior. In addition to internal states and drives, actions are also determined by the incentive properties of our environment. Through experience, many objects come to be associated with appetitive or aversive motivational properties that guide our interaction with these objects in daily life. In contrast, research on the effects of motivation on attention or memory commonly employs artificial stimuli for which motivational relevance is learned during the experiment. Some studies use real objects or scenes (Hickey et al., 2015), but pre-existing motivational value is rarely considered. Previous databases of emotional and motivational value generally present decontextualized objects on a blank background, such as food (Blechert et al., 2014; Miccoli et al., 2014). Research on the neural and behavioral effects of appetitive and aversive motivation, such as investigations of its effects on attention, memory and choice, could therefore benefit from a comprehensive database of the motivational value assigned to everyday objects in more complex scenes.

Motivational stimuli and internal motivational states serve as inducements to action by affecting the direction, intensity and duration of a set of actions. The most basic and longstanding distinction that characterizes motivated behavior distinguishes actions of approach and avoidance that are elicited by appetitive and aversive factors, respectively (Thorndike, 1911; Watson, 1913; Skinner, 1938; Higgins, 1997). Motivation can thus direct actions toward or away from objects associated with high or low subjective utility (see for example Elliot, 2006; Braver et al., 2014). Although this association suggests that motivational drive is related to the hedonic experience of action outcomes and thus to emotional experience, the two concepts can be distinguished (Berridge and Robinson, 1998). Both humans and non-human animals may display motivated actions in the absence of hedonic reactions to the outcome (referred to as ‘liking’), and conversely experience hedonic reactions in the absence of motivational drive (referred to as ‘wanting’), and the two states have been suggested to involve different brain systems (for a review, see Berridge, 2015). For research using natural stimuli, it is therefore important to separately control for the motivational drive elicited by objects and object-related emotional reactions. An additional, largely separate aspect is the frequency with which objects are encountered in daily life. Lower frequency may lead to reduced recognizability, which would make the ratings less interpretable.

Studies using realistic motivational stimuli have been scarce. Here we introduce a new picture database, Motivational Objects in Natural Scenes (MONS). 805 objects were taken from newly photographed natural scenes intended for experimental use. Objects have been shown to elicit strong motivational effects in consumer settings (e.g., Elliott, 1998; Seva et al., 2007; Cohen et al., 2008; Alba and Williams, 2013), findings that are supported by recent fMRI evidence on product-related decision making (e.g., Knutson et al., 2007; Chib et al., 2009; Levy et al., 2011).

**FIGURE 1:**
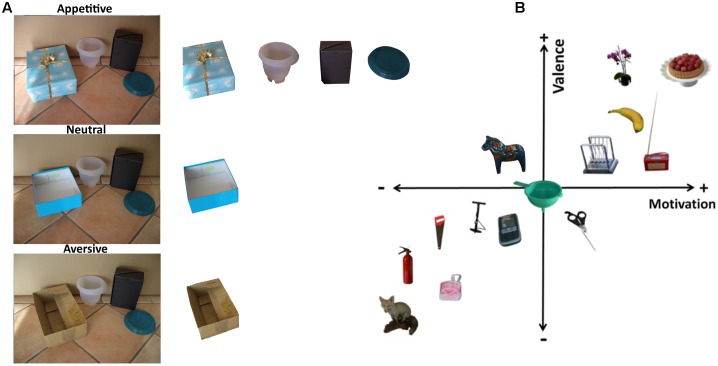
**(A)** Example of three versions of a realistic scene from which the objects were cut out. In each version of the scene, one object was replaced that varied on the level of motivational value. On the top the appetitive version (a present), in the middle the neutral version (a shoe box), and on the bottom the aversive (an old carton) version. All unique objects were cut out from the scene and rated separately. **(B)** Example Motivational Objects in Natural Scenes objects on the motivation and valence dimensions for the objects in isolation ratings. Note, the object locations do not reflect exact values, but objects are spaced for demonstrational purposes.

These studies and existing databases mostly used isolated objects, motivating the natural-scene database presented here. The term natural scenes is often used to refer to realistic scenes that we could encounter in every-day life (Hart et al., 2013; Marx et al., 2014; Stoll et al., 2015). At the same time, low-level physical properties of a scene influence visual processing and guidance of attention (Awh et al., 2012). Hence, we aimed to control for low-level visual properties by photographing each scene at least three times, each time replacing one object (“critical object”) in the scene to create versions with varying motivational value (see **Figure 1** for an example). Although scenes photographed in this way may still look slightly artificial as compared to our typically cluttered environment, they are still composed of real objects. Indeed, such scenes with nicely arranged objects are actually quite common in everyday life, for example in shop displays. The scene creation procedure also allowed us to maintain a high level of visual similarity between scenes and objects while varying their motivational properties. In addition to the critical object, each scene contains one to six additional neutral objects. On the basis of the motivational ratings, the images were categorized as appetitive, neutral, and aversive. We acquired ratings for the objects isolated from the scenes presented on a white background using online questionnaires and separate ratings for the critical objects presented in their scene context. Each object was rated on three scales intended to measure different aspects of motivation and three scales measuring possible confounding factors. In addition, we obtained ratings for the entire scenes.

Because of the diversity of objects in the database, we designed three scales to capture the main aspects of motivation. Although the three scales were expected to measure a single dimension – motivation – we reasoned that the motivational properties of some objects might be captured better by only one or two of the scales. The action component that defines motivation was therefore measured by two scales assessing the wish to interact with an object (Interaction scale) and whether to approach or avoid (Approach/Avoid scale) the object. Note that the term interaction may be broadly interpreted, reflecting the typical action associated with an object (e.g., eating in the case of food, and viewing in the case of a painting). Although the two scales overlap in many cases, ratings may differ for some objects (for example, one may not wish to interact with a musical instrument if one cannot play it, but one may wish to approach it to hear others play). Related to this action component of motivation is the distinction between objects that are typically associated with passive or active behavior. A clear perception-action link, also referred to as affordance, will probably lead to approach and interaction behavior (Gibson, 1979). No affordance scale was included in order to keep the number of ratings manageable for subjects, but our interaction and approach/avoidance scales may have partly captured this concept.

The third motivational scale addressed the component of wanting (Berridge et al., 2009) by measuring how much participants desired to own the object (Desire to Own scale). For many objects, this scale will be correlated with the other two scales. However, in some cases the scales may differ (e.g., one may wish to interact with a park bench by sitting on it, but may not wish to own it; see **Table 1** for all scale questions).

**Table 1:** Rating questions per scale for object ratings (Part I and Part II) and scene ratings (Part III) on 7-point Likert scales.

	Question	1-, 4- and 7-point label
**Object ratings**		
*Motivational scales*		
(1) Approach/Avoid	Do you want to approach/avoid the object?	*Avoid-Neutral-Approach*
(2) Desire to Own	How much would you like to own the object?	*Not at all-Neutral-Very much*
(3) Interaction	How much would you like to interact with the object?	*Not at all-Neutral-Very much*
*Control scales*		
(4) Arousal	Does this object make you calm or aroused?	*Calm-Neutral-Aroused*
(5) Valence	Does this object elicit any positive or negative emotions?	*Negative-Neutral-Positive*
(6) Recognizability	Do you recognize the object?	*Not at all-Neutral-Very familiar*
**Scene ratings**		
(1) Approach/Avoid	Do you want to approach/avoid the place?	*Avoid-Neutral-Approach*
(2) Exploration	Do you want to explore the scene?	*Not at all-Neutral-Very much*
(3) Spending time	How much would you value spending time in the place?	*Not at all-Neutral-Highly*
(4) Valence	Does this scene elicit any positive or negative emotions?	*Negative-Neutral-Positive*
(5) Arousal	Does this scene make you calm or aroused?	*Calm-Neutral-Aroused*
**Quality rating**		
Quality	Please rate the image quality	*Very poor-Fair-Excellent*


We aimed to control for the affective properties of the objects. The selected objects were intended to vary more widely in motivational value than in their emotional impact. Widely accepted dimensional theories of emotion generally divide the affective space into the two dimensions valence and arousal (Russell, 1980; Lang et al., 2008). Two scales measuring Valence and Arousal were therefore included in the ratings. An additional scale was included to control for recognizability of the objects (see for example Martin et al., 2001).

The purpose of the current database is to provide vision researchers and cognitive neuroscientists with a controlled stimulus set consisting of realistic natural scenes and every-day objects. The database will allow scientists to use stimuli with controlled ratings on motivation, valence, arousal, or recognizability when one of these dimensions is relevant in their experiment. The database can be used to select motivationally neutral stimuli, for example to exclude motivational influences in memory tasks, as well as to select appetitive or aversive stimuli, for example in studies of visual attention. Vision scientists may be especially interested in our natural scene set, as the scenes were controlled in terms of visual characteristics (see above), and we provide exact object locations within the scenes.

To support a range of experimental uses, we collected ratings on the isolated objects, on the objects in the scenes and on the scenes as a whole. For each set of ratings, we conducted a principal component analysis. Please note: Throughout the text, we will refer to the motivational categories as appetitive, neutral and aversive, in contrast to the valence ratings, which we will refer to as positive and negative or low and high in valence.

## General Experimental Procedures

### Stimulus Creation

For this database, motivational and neutral objects in natural scenes were photographed at least three times, each time replacing one object (“critical object”) that was intended to vary in motivational value. The scenes were controlled such that objects were mostly decentralized, objects only overlapped minimally and scenes contained between 2 and 7 objects. Each triad of scenes was photographed from the same visual angle, keeping lighting conditions as similar as possible. In each version of a triad of scenes one object varying in intended motivational value was replaced, aiming to create versions that differed in terms of motivational value. All other objects in the scenes were intended to be neutral. We selected objects to vary on motivational value, and later subdivided the stimuli into motivational categories on basis of the ratings. We did not include animate objects, objects with human face depictions, or objects with large texts, as these aspects are all known to draw attention (Cerf et al., 2008, 2009), irrespective of motivation. Small texts were later removed using image manipulation software (see below).

Part I reports ratings of all objects in isolation (cut out of the scenes) on three scales intended to measure motivation (Interaction, Desire to Own, and Approach/Avoid), two emotional scales measuring valence (negative to positive) and arousal (calming to exciting), and a scale measuring recognizability (unrecognizable to familiar). Part II reports ratings of the critical objects in the scenes on all scales except Recognizability, as we expected that the objects would be more easily recognized in their original context. Part III reports ratings of the entire scenes on motivation (Approach/Avoid, Spending Time and Exploration), arousal and valence. For some scenes, more than three versions were created, but only three were selected for the scene ratings, in order to create balanced questionnaire versions (Part III). Some objects were extracted from scenes that were not included in the final version of the database. For completeness, these unrated scenes can be found in the online database (scene numbers > 107).

In addition to the 107 triads of scenes eventually used in Parts II (objects in scenes ratings) and III (scene ratings), an additional 40 critical objects were presented in Part I, yielding a total of 805 objects to be investigated in isolation. Objects were cut out from the photographed scenes and all legible text was removed using the GNU Image Manipulation Program 2^1^. The cutout objects are presented on a white background and rescaled to fit in a 1000 pixel × 1000 pixel image preserving aspect ratio.

To verify that the background objects, which were intended to be neutral, are not perceived as strongly motivational, we quantified their deviance from neutrality. A non-neutrality measure (also reported in the online documentation) was created by summing over the absolute difference of ratings for neutral, non-critical objects in the scene when these deviated from the neutral range (3.5–4.5). To support a range of experimental uses of the database, separate deviance measures were calculated for the mean motivation, valence and arousal scales.

All objects, scenes, and rating data can be downloaded free of charge at doi: 10.5281/zenodo.883110. The exact locations of the objects in the scenes are included in the form of coordinates of bounding boxes containing the objects.

### Participant Recruitment

Inclusion criteria were age 18–45 years, no history of neurological or psychiatric illnesses (as subjectively reported). Only participants reporting to have good or excellent English skills could participate. The study was approved by the ethics committee of the Department of Psychology and Sports Science at the Justus Liebig University, Giessen. All participants gave informed consent by checking a box. Respondents were recruited via mailing lists of several universities in Europe (including Germany, the Netherlands, and the United Kingdom), social media, and online message boards, creating a diverse group of respondents. After completing the questionnaire participants could enter a draw to win one of forty 25 Euro (or equivalent) gift vouchers for an online store (Part I) or one of twenty 20 Euro (or equivalent) gift vouchers (Parts II and III). Data were collected through an internet-based survey system (Leiner, 2014). Participants could pause the questionnaire to complete it a later convenient time. Completion of the questionnaire took around 35–45 min for objects in isolation (Part I), 25–30 min for objects in scenes (Part II), and 15–25 min for scenes (Part III).

## Part I: Ratings of Objects in Isolation

### Methods

#### Participants

The questionnaire was started by 904 volunteers, of whom 439 were eligible based on the inclusion criteria *and* filled in the questionnaire. After reading the study information on the first pages of the questionnaire, 51 participants decided to not participate and did not give informed consent by checking the box. Also, our inclusion criteria were quite strict. The age range of 18–45 years led to the exclusion of 31 participants. In addition, we excluded all participants with a self-reported history of or a current psychiatric illness (*n* = 58). The inclusion on basis of English skills also led to the exclusion of a relatively large number of subjects (*n* = 152), as we promoted our questionnaire among undergraduate students in Germany. Also, non-disclosure on any of these inclusion criteria led to exclusion (*n* = 173). Non-eligible volunteers were directed to the end of the questionnaire and could not fill in the questionnaire. Eight participants were excluded because of poor data quality (>80 min to complete the questionnaire or no variation in answers). Four hundred and thirty-one participants [257 female; age 18–45 years, mean = 25.6, standard deviation = 4.6; highest completed education: Range from Secondary/High school (level 2) to Doctorate/Professional degree (level 6), median = Bachelor degree (level 4)] were included in the analyses. Each participant filled in one of 12 versions of the questionnaire consisting of randomly chosen unique objects. The number of participants and participant details per version of the questionnaire can be found in **Table 2**. Data from unfinished questionnaires were included in the analyses.

**Table 2:** Participant details per questionnaire type and version.

Questionnaire type and version	*n*	Female/male	Age range	Mean age in years	Education range	Mean Education
**Objects in isolation**						
Version 1	38	24/14	18–44	25.4	2–6	3.6
Version 2	25	18/7	20–35	25.9	2–6	4.0
Version 3	38	16/22	18–37	25.5	2–6	3.5
Version 4	47	28/19	18–41	25.4	2–6	3.7
Version 5	43	22/21	19–45	26.7	2–6	4.2
Version 6	45	25/20	19–44	26.4	2–6	3.6
Version 7	40	29/11	19–35	25.8	2–6	4.1
Version 8	35	21/14	18–43	26.2	2–6	3.8
Version 9	32	21/11	18–39	25.6	2–6	3.6
Version 10	39	21/18	18–36	25.5	2–6	3.6
Version 11	26	11/15	18–36	26.0	2–6	3.7
Version 12	23	21/2	18–28	22.5	2–5	3.5
Subtotal	431	257/174	18–45	25.6	2–6	3.7
**Objects in scenes**						
Version 1	29	19/10	18–40	25.3	2–6	3.6
Version 2	40	29/11	18–39	24.1	2–6	3.4
Version 3	26	20/6	19–34	23.9	2–6	3.5
Version 4	34	22/12	18–39	25.1	2–6	3.6
Version 5	31	25/6	18–45	24.0	1–6	3.3
Version 6	35	22/13	18–33	23.5	2–6	3.1
Subtotal	193	137/58	18–45	24.3	1–6	3.4
**Scenes**						
Version 1	29	22/7	18–31	22.5	2–6	2.9
Version 2	24	19/5	19–45	25.7	2–6	3.3
Version 3	27	21/6	18–34	24.0	2–5	3.4
Version 4	30	21/9	18–43	24.1	2–6	3.4
Version 5	29	20/9	18–41	23.4	2–6	3.3
Version 6	24	18/6	19–35	23.9	2–6	3.4
Subtotal	163	121/42	18–45	23.9	2–6	3.4
Total	787	515/274	18–45	24.6	1–6	3.5


*Education: Level 1 = Primary/Elementary school; Level 2 = Secondary/High school; Level 3 = Some form of additional education (e.g., college or technical institute); Level 4 = Bachelor’s degree; Level 5 = Master’s degree; Level 6 = Doctorate/Professional degree.*

Twelve versions of the questionnaire were created, each containing 71 objects, except for the eleventh and twelfth questionnaires, which consisted of 45 and 50 objects, respectively. In total, ratings were obtained for 805 objects. For presentation in the questionnaire, the images were further rescaled to 300 pixels × 300 pixels. All objects were rated on six 7-point Likert scales. To the authors’ knowledge, no scales currently exist that aim to measure motivational value of natural scenes or realistic objects from a range of categories (food and non-food), therefore we constructed three scales for this purpose: (1) Desire to Own (“How much would you like to own this object?”); (2) Approach/Avoid (“Do you want to approach or avoid the object?”); (3) Interaction (“How much would you like to interact with the object?”). The scales went from “Not at all” to “Very much.” The other scales measured (4) Arousal (“Does this object make you calm or aroused?”) from “Calm” to “Aroused”; (5) Valence (“Does this object elicit any positive or negative emotions?”) from “Negative” to “Positive,” and (6) Recognizability (“Do you recognize the object?”) from “Not at all” to “Very Familiar.”

#### Analyses

To investigate how the different scales (Desire to Own, Approach/Avoid, Interact, Arousal, Valence, and Recognizability) relate, we performed a principal component analysis (PCA). To reduce possible effects of individual bias, prior to the analyses described in this section (ICC and PCA), ratings were z-transformed per participant, separately for each scale (Desire to Own, Approach/Avoid, Interact, Arousal, Valence, and Recognizability). To check whether objects were similarly rated by the different raters who used the same questionnaire, we calculated the intraclass correlation coefficient (ICC) separately for each scale, using the R package R3.1.0 (version 0.84; R Core Team, 2014). For all of the 72 combinations of questionnaire (12) and scale (6), the ICC was significantly larger than 0 (range: 0.024–0.308; all *p*s < 0.003). Therefore, data for subsequent analyses was aggregated over all raters for the principal component analysis (PCA). After the first rotation, the number of components to extract was determined using visual inspection of the scree plot of the eigenvalues per scale. In the second step of the PCA, a varimax rotation was used (Kaiser, 1958). Since results from the PCA showed that the three motivational scales (Desire to Own, Approach/Avoid, and Interaction) correlated strongly, these scales were taken together in further analyses by calculating the mean motivation rating over these scales. Although valence also loaded highly on the same factor, it was not taken together with the motivational scales as the current database focuses on motivation and databases with emotional stimuli are already available (e.g., IAPS; Lang et al., 2008).

*Post hoc* we created three categories of motivational objects. Objects for which the mean motivational rating was ≤3.5 were categorized as aversive, objects rated 3.5–4.5 were categorized as neutral, and objects rated ≥4.5 were categorized as appetitive (see **Table 3** for details on the *post hoc* motivational categories per scale). As this *post hoc* division created categories unequal in size, we also provide a division in quintiles in the online documentation files. The division in quintiles resulted in two levels of aversive (I and II), one level of neutral (III), and two levels of appetitive (IV and V) categories, and may be used if a more fine-grained division in motivational categories is preferred.

**Table 3:** Motivational object category details for the ratings of objects in isolation (Part I).

Motivational object category	*n*	Range	Mean (*SD*)
**Mean Motivation**			
Aversive	136	1.30–3.49	2.97 (0.45)
Neutral	385	3.51–4.49	4.03 (0.27)
Appetitive	284	4.50–6.26	4.97 (0.38)
*Motivation Total*	*805*	*1.30–6.26*	*4.18 (0.37)*
**Valence**			
Aversive	136	1.68–4.83	3.63 (0.49)
Neutral	385	3.25–5.40	4.06 (0.42)
Appetitive	284	3.37–6.02	4.64 (0.49)
*Valence Total*	*805*	*1.68–6.02*	*4.19 (0.47)*
**Arousal**			
Aversive	136	2.61–5.50	3.74 (0.50)
Neutral	385	2.91–4.89	3.67 (0.39)
Appetitive	284	2.55–5.49	3.83 (0.50)
*Arousal Total*	*805*	*2.55–5.50*	*3.74 (0.46)*
**Recognizability**			
Aversive	136	2.16–5.63	4.64 (0.70)
Neutral	385	2.17–6.24	4.88 (0.62)
Appetitive	284	4.19–6.03	5.21 (0.29)
*Recognizability Total*	*805*	*2.16–6.24*	*4.96 (0.58)*


*Objects were categorized *post hoc* on the basis of mean Motivation, collapsing over the Approach/Avoid, Desire to Own, and Interaction scales: Mean motivation ≤ 3.5: aversive; mean motivation 3.5–4.5: neutral; mean motivation ≥ 4.5: appetitive. Ratings are reported for the aversive, neutral, and appetitive motivational *post hoc* categories.*

Since the objects were cut out of pictures of scenes, the critical objects were not always in focus, and sometimes partly occluded, possibly affecting the image quality. As image quality might have affected the ratings, we also obtained subjective quality ratings (“Please rate the image quality”) on a 7-point scale from “Very poor” to “Excellent.” To obtain a general quality rating, no concrete instructions were given concerning the rating criteria. Two quality questionnaires were composed, one with 400 and the other with 405 objects, that were filled out by new participants. As subjective image quality ratings may be affected by motivational modulation, we additionally calculated an objective image quality assessment (IQA; Mittal et al., 2013).

### Results

More females (*N* = 257) than males (*N* = 174) filled in the questionnaire, and the ratio of female/male participants was different for the different versions, χ^2^(11, *N* = 431) = 24.83, *p* = 0.010. In the online documentation, we report mean ratings for males and females separately, as well as the mean ratings across gender. Details on the *post hoc* motivational object categories can be found in **Table 3**. In total, 136 objects were categorized as aversive (mean motivational rating ≤ 3.5), 385 as neutral (mean motivational rating 3.5–4.5), and 284 as appetitive (mean motivational rating >4.5), making a total of 805 objects.

#### PCA Analyses

**FIGURE 2:**
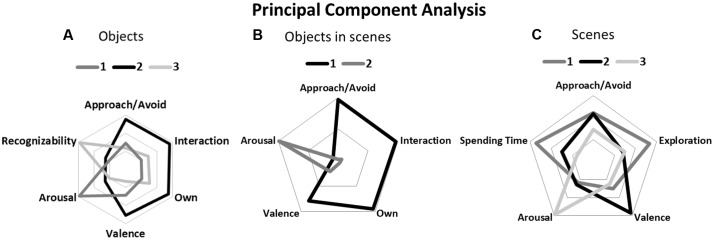
Component loadings are shown for each scale. Part 1 **(A)**: Ratings for objects in isolation for the motivational scales Approach/Avoid, Interaction, and Desire to Own all loaded highly on component 1 together with valence. Arousal loads highly on component 2, while Recognizability loads highly on component 3. Part II **(B)**: Ratings for objects in scenes. The motivational scales (Approach/Avoid; Interaction; Desire to Own) all loaded highly on component 1 together with valence. Arousal loaded highly on component 2. Part III **(C)**: Scene ratings for the Approach/Avoid, Spending Time, Exploration, Valence, and Arousal scales. Approach/Avoid, Exploration, and Spending Time loaded highly on component 1, Valence and Approach/Avoid on component 2, and Arousal on component 3.

Ratings were collapsed over raters for the PCA analyses. A PCA analysis was performed on the *z*-transformed ratings to investigate the underlying structure of the six scales. Visual inspection of the scree plot after the initial rotation showed that the curve dropped off at two points (after component 1 from eigenvalue 3.28 to 1.07 for component 2, and from component 3 to 4 from 0.83 to 0.47). Since the eigenvalues of component 2 and 3 were larger than or close to 1 (1.07 and 0.83, respectively), and the eigenvalue for component 4 dropped substantially (0.46), three components were extracted in the second step of the analysis. Varimax rotation was performed for three extracted components. All three motivational scales (Desire to Own, Approach/Avoid, Interaction) and Valence loaded highly on component 1, which explained 54.62% of the variance. Arousal loaded highly on component 2, explaining 17.80% of the variance (72.49% cumulatively). Recognizability loaded highly on component 3, explaining 13.77% of the variance (86.27% cumulatively; see **Figure 2A** for the component loadings for each scale), confirming their independence from the motivational scales.

#### Image Quality

Thirty (24 female; age range = 18–39; age mean = 23.0; education range = 2–4; education median = 3) and 22 (18 female; age range = 18–31; age mean = 23.2; education range = 2–5; education median = 3) newly recruited participants completed the two image quality questionnaires. Motivational category affected the quality ratings, *F*(2,802) = 3.60, *p* < 0.028, η^2^ = 0.01. *Post hoc* comparisons with Bonferroni correction for multiple comparisons were performed to compare the motivational categories. Appetitive objects received higher ratings (mean = 3.87; *SD* = 0.98) than aversive objects (mean = 3.61; *SD* = 1.02), *t*(418) = 2.52, *p* = 0.012, and subjective quality for appetitive and neutral objects did not differ (mean = 3.73; *SD* = 0.95), *t*(667) = 1.81, *p* = 0.071. Quality ratings were similar for aversive and neutral objects (*p* = 0.196). Motivational category also affected the objective image quality assessment, *F*(2,802) = 6.61, *p* = 0.001, η^2^ = 0.002. In line with the subjective ratings, appetitive objects were calculated as being of higher quality (mean = 7.58; *SD* = 2.74) than aversive objects (mean = 8.46; *SD* = 3.66), *t*(418) = 2.75, *p* = 0.006 (note, higher image quality assessment values reflect lower quality). In addition, neutral objects were estimated to have higher quality than aversive objects (mean = 7.53; *SD* = 2.14), *t*(519) = 3.56, *p* < 0.001. The objective quality assessment was similar for appetitive and neutral objects (*p* = 0.786).

To further investigate whether the quality ratings were affected by other factors, we calculated a correlation between the quality measures and the recognizability rating. The subjective ratings showed a positive correlation with recognizability, *r*(805) = 0.12, *p* = 0.001, but no correlation was found for the objective quality measure (*p* = 0.401), suggesting that recognizability affected subjective perception of quality. Both subjective and objective quality measures can be found in the object ratings documentation file.

## Part II: Ratings of Objects in Scenes

### Methods

#### Participants

The questionnaire was started by 510 volunteers, of whom 195 eligible participants (137 female; age 18–45 years, mean = 24.3, standard deviation = 5.0; education range: 1–6; education median = 3) filled it in and were included in the analyses. Each participant filled in one or two of six versions of the questionnaire including unique versions of the triads of scenes containing one relevant object, such that the same scene was not repeated for a particular participant. Participant details per version of the questionnaire can be found in **Table 2**.

#### Procedure

Ratings were obtained for the critical objects; that is, for the object in a scene that is varied across the triad. In contrast to the first object-rating experiment, objects were now presented in the scenes in which they were originally photographed rather than in isolation. The relevant object that required rating was highlighted by a box with black/white dashed lines. Participants filled in up to two versions of the questionnaire that each contained 60 (first three questionnaires) or 56 objects (last three versions). In total, ratings were obtained for 348 unique objects in 116 different scenes. Nine scenes were excluded due to poor image quality, resulting in a total of 107 scenes that were included in the analyses. The scenes were all presented on a white background and rescaled to 750 pixels × 562 pixels. The objects in the scenes were rated on the same scales as the objects in isolation except for the Recognizability scale, as we expected that the objects would be more easily recognized in their original context.

#### Analyses

To validate findings from the objects-in-isolation ratings, a PCA was performed using the same procedures. Since results from the PCA again showed that the three motivational scales (Desire to Own, Approach/Avoid, and Interaction) correlated strongly, these scales were taken together in further analyses as in Part I. To confirm that the ratings for the objects in isolation were not affected by reduced recognizability due to out of context presentation, nor ratings for the objects in scenes affected by their context, correlations were calculated between the ratings per scale.

### Results

**Table 4** shows details about the *post hoc* motivational categories per scale. In total, 104 objects were categorized as aversive (mean motivational rating ≤ 3.5), 127 as neutral (mean motivational rating 3.5–4.5), and 90 as appetitive (mean motivational rating > 4.5), making a total of 321 objects in scenes. Note that the total number of objects is lower than in Part I, as ratings were only obtained for the ‘critical’ objects, that is, the objects that were replaced in the scene and that were intended to vary in motivational value.

**Table 4:** Motivational object category details for the ratings of the objects in scenes (Part II).

Motivational object category	*n*	Range	Mean (*SD*)
**Mean Motivation**			
Aversive	104	1.16–3.49	2.67 (0.60)
Neutral	127	3.52–4.48	3.97 (0.26)
Appetitive	90	4.52–6.12	5.14 (0.40)
*Motivation Total*	*321*	*1.16–6.12*	*3.88 (0.42)*
**Valence**			
Aversive	104	1.85–4.80	3.70 (0.55)
Neutral	127	3.19–4.88	3.96 (0.38)
Appetitive	90	3.58–5.63	4.65 (0.46)
*Valence Total*	*321*	*1.85–5.63*	*4.08 (0.46)*
**Arousal**			
Aversive	104	2.95–5.32	3.83 (0.44)
Neutral	127	2.90–4.55	3.64 (0.34)
Appetitive	90	3.04–5.68	4.05 (0.46)
*Arousal Total*	*321*	*2.90–5.68*	*3.82 (0.42)*


*Objects were categorized *post hoc* on the basis of mean Motivation, collapsing over the Approach/Avoid, Desire to Own, and Interaction scales: Mean Motivation ≤ 3.5: aversive; mean Motivation 3.5–4.5: neutral; mean Motivation ≥ 4.5: appetitive. Ratings are reported for the aversive, neutral, and appetitive motivational categories.*

#### PCA Analyses

Visual inspection of the scree plot after the initial rotation showed that the curve again dropped off at two points (after component 1 from eigenvalue 3.47 to 0.98 for component 2, and from component 2 to 3 from 0.98 to 0.42). Using the same criteria as for the ratings of objects in isolation, we chose to extract two components in the second step of the analysis. Varimax rotation replicated the findings for isolated objects, as the three motivational scales (Desire to Own, Approach/Avoid, Interaction) and Valence loaded highly on component 1, explaining 69.38% of the variance. Arousal loaded highly on component 2, explaining 19.54% of the variance (88.93% cumulatively; see **Figure 2B** for the component loadings for each scale), confirming its independence from the motivational scales.

#### Correlations between Ratings for Objects in Isolation and Objects in Scenes

**FIGURE 3:**
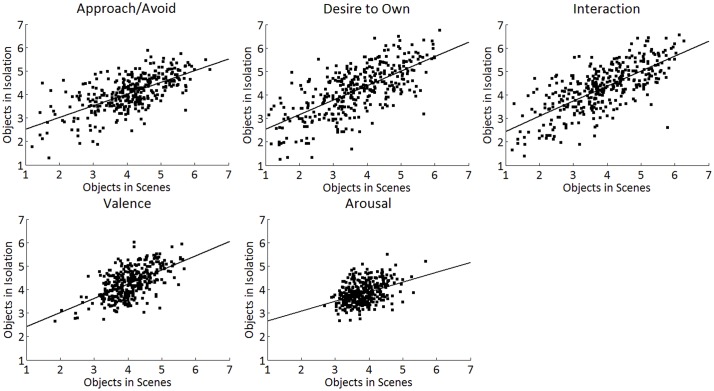
Correlations between the objects-in-scenes ratings and the objects-in-isolation ratings for the different motivational scales (Top): Approach/Avoid, Desire to Own, and Interaction. Note that scores on the non-motivational control scales Valence and Arousal (Bottom) show less variance, as intended.

For the motivational scales Approach/Avoid, Interaction, and Desire to Own, positive correlations were observed between the ratings for the objects in the isolation and in the scenes: *r*(321) = 0.66, *p* < 0.001, *r*(321) = 0.71, *p* < 0.001, and *r*(321) = 0.68, *p* < 0.001, respectively. A positive correlation was also found for the valence ratings, *r*(321) = 0.59, *p* < 0.001 and arousal ratings, *r*(321) = 0.41, *p* = 0.001. These correlations are depicted in **Figure 3**.

## Part III: Scene Ratings

### Methods

#### Participants

The questionnaire was started by 426 participants, of whom 164 were eligible. One participant was excluded because of a report of technical problems. 163 participants were included in the analysis (121 female; age 18–45 years, mean = 23.9, standard deviation = 5.3; highest completed education range from 2 to 6; education median = 4). Each participant filled in one of 6 versions of the questionnaire consisting of 116 scenes. Participant details per questionnaire version can be found in **Table 2**.

#### Procedure

Ratings were obtained for the entire scenes rather than specific objects. Six versions of the questionnaire were created, the first three each containing 60 scenes, and the last three containing 56 scenes. Ratings were obtained for three versions of 116 scenes (348 in total; but note that nine triads were excluded due to poor image quality, resulting in a total of 321 scenes). Each participant did not rate more than one version of the same scene. The scene size at presentation was 750 pixels × 562 pixels. All scenes were rated on five 7-point Likert scales. For the ratings of the entire scenes, we could not use the same set of scales as for the objects because interacting with an object (Interaction), owning an object (Desire to Own) and being familiar with an object (Recognizability) do not directly transfer to whole scenes. Again, three scales related to motivational value were created: Approach/Avoid, Spending Time and Exploration. The last two were intended to replace Interaction. The other scales measured Arousal and Valence. See **Table 1** for the exact questions per scale. Scenes were subdivided *post hoc* into motivational categories on basis of the motivational ratings for that scene (collapsing across Exploration, Approach/Avoid and Spending Time, as these were found to load on the same PCA component as expected).

#### Analyses

To investigate how the different scales (Approach/Avoid, Spending Time, Exploration, Arousal, and Valence) relate, we again performed a PCA, following the same procedures as for the objects. The PCA results showed that Exploration, Approach/Avoid and Spending Time correlated strongly.

### Results

Although the database was created to obtain motivation ratings for specific everyday objects, we also obtained ratings for the scenes to provide fully controlled items for use in a range of experimental settings. The images were not created with the aim of making the whole scenes motivational; however, we expected that the scene ratings would be affected by the motivational value of the critical object. Again, we divided the scenes *post hoc* into aversive, neutral, and appetitive based on the mean motivational ratings. For this, the motivational scales Approach/Avoid, Exploration, and Spending Time were used as these scales loaded on one PCA factor.

**Table 5** shows the details for the motivational categories for the different scales. In total, 52 scenes were categorized as aversive (mean motivational rating ≤ 3.5), 218 as neutral (mean motivational rating 3.5–4.5), and 51 as appetitive (mean motivational rating > 4.5), making a total of 321 scenes.

**Table 5:** Motivational object category details for the ratings of the scenes (Part III).

Motivational object category	*n*	Range	Mean (*SD*)
**Mean Motivation**			
Aversive	56	2.18–3.50	3.17 (0.32)
Neutral	214	3.52–4.49	3.98 (0.26)
Appetitive	51	4.53–5.58	4.77 (0.24)
*Mean Motivation Total*	*321*	*2.18–5.58*	*3.96 (0.29)*
**Valence**			
Aversive	56	2.57–4.64	3.74 (0.39)
Neutral	214	2.93–5.00	3.86 (0.36)
Appetitive	51	3.65–5.76	4.53 (0.43)
*Valence Total*	*321*	*2.57–5.76*	*3.96 (0.38)*
**Arousal**			
Aversive	56	2.91–4.52	3.70 (0.35)
Neutral	214	3.00–5.38	3.67 (0.33)
Appetitive	51	3.00–4.93	3.92 (0.42)
*Arousal Total*	*321*	*2.91–5.38*	*3.72 (0.34)*


*Scenes were categorized *post hoc* on basis of the mean motivational ratings, collapsing over the Approach/Avoid, Spending Time and Exploration scales. Mean Motivation ≤ 3.5: aversive; mean Motivation 3.5–4.5: neutral; mean Motivation ≥ 4.5: appetitive. Ratings are reported for the aversive, neutral, and appetitive motivational categories.*

#### PCA Analyses

A PCA analysis was performed for the scene ratings using the same procedures as for the objects. The scree plot dropped off at two points, after component 1 going from an eigenvalue of 2.83 to 0.94 for component 2, and after component 3 to 4, going from an eigenvalue of 0.66 to 0.37. Using the same criteria as for the previous PCA analyses, three components were included in the Varimax rotation. Approach/Avoid, Exploration, and Spending Time loaded highly on the first component, explaining 56.47% of the variance. Therefore, these scales were used to subdivide the scenes in terms of motivation (aversive, neutral, and appetitive). Valence and to a lesser extent Approach/Avoid loaded highly on component 2, explaining an additional 18.86% (75.33 cumulatively), while Arousal loaded highly on component 3, explaining 11.15% of the variance (86.45% cumulatively). See **Figure 2C** for the component loadings for each scale.

#### Correlations between Object Ratings and Scene Ratings

To check whether the scene ratings reflected the motivational value of the objects in them, we correlated the mean approach/avoid rating of all objects in a scene with its general approach/avoid scene rating. This scale was selected, as – unlike for the other scales – the instruction in object and scene rating was most similar. These mean object ratings per scene can be found in the mean object ratings file of the database. A positive correlation was observed, *r*(321) = 0.22, *p* < 0.001, suggesting that the scene ratings indeed reflect the motivational value of the depicted objects.

## Discussion

The present study introduces MONS, a standardized database of natural appetitive, aversive and neutral objects and scenes. For this purpose, motivational and neutral objects in natural scenes were photographed at least three times, each time replacing one critical object varying in motivational value. All objects were rated in isolation (cut out of the scenes), and the critical objects were additionally rated in the scenes on three scales intended to measure motivation (Interaction, Desire to Own, and Approach/Avoid) and two additional scales measuring valence (negative to positive) and arousal (calming to exciting). In addition, the objects in isolation were also rated on recognizability (novel to familiar). Finally, ratings were obtained for the entire scenes measuring motivation (Approach/Avoid, Spending Time and Exploration), Arousal and Valence.

All ratings were subjected to separate PCAs for objects in isolation, objects in scenes, and scenes. As expected, Arousal measured independent constructs for all ratings. Recognizability ratings, which could only be obtained for the objects in isolation, were also confirmed to be independent of the other scales. As predicted, for both types of object ratings the three motivational scales all loaded highly on one component, indicating that they measure a single underlying construct, presumably motivational value. Similarly, all motivational scales (Approach/Avoid, Exploration, and Spending Time) for the scene ratings loaded highly on one component.

Valence, however, also loaded strongly on this component in both object rating experiments, and loaded highly on a component on which the motivational Approach/Avoid scale also loaded highly in the scene rating experiment, suggesting that motivation covaried with the affective reactions elicited by the objects and scenes. This conflation of valence and motivation is not unexpected, as motivational drive is related to the hedonic experience of action outcomes and thus to emotional experience. Nonetheless, the two concepts can be distinguished. Valence is more strongly linked to ‘liking’ and ‘disliking,’ whereas motivation is more strongly associated with ‘wanting’ (Berridge, 2009; Berridge et al., 2009). Previous studies indicate that objects can elicit purchase motivation (e.g., Elliott, 1998; Seva et al., 2007; Cohen et al., 2008; Alba and Williams, 2013), mediated on a neural level by the motivational system (Knutson et al., 2007; Chib et al., 2009; Levy et al., 2011). The current confound between motivational ratings and valence may be a natural consequence of the connection between emotion and motivation in the real world. However, this association was mostly found in the PCA and is only weakly reflected in the category means, where both aversive and neutral motivational objects were rated in the neutral range on valence and appetitive objects were rated as only slightly above the neutral range on valence. Moreover, although there was a general link between valence and motivation, individual objects deviated from this general pattern. Researchers interested in separating the effects of valence and motivation could therefore select those images in which valence and motivation ratings differed the most. For example, a medicine bottle, bag of cat litter and banana peel were rated as neutral on valence, but quite aversively on motivation. Vice versa, a kitchen knife, toilet paper and an umbrella were rated quite low on valence, but rather high on motivation. These differences demonstrate that affective impact and motivational drive can be distinguished for everyday objects.

We also addressed the possibility that the ratings were affected by image quality. Both subjective quality reports and objective quality assessment measures showed that the appetitive objects have a higher quality than the aversive objects. Possibly, the quality of the appetitive objects was indeed coincidentally higher, however, it is also possible that motivation affected the quality ratings, maybe through an attentional mechanism. Another possibility is that recognizability affected the subjective quality ratings, as a positive correlation between recognizability and the subjective quality ratings, but not the objective quality assessment was found. Maybe participants based their quality estimate partly on whether they recognized the object. Users of the database may want to use either the quality or recognizability measures when selecting images for their research, or include these measures in their analyses. The object ratings were not strongly affected by the presentation of the objects in isolation or in the scenes, supported by the consistent PCA results and by significant correlations between object ratings in isolation and object ratings in the scenes. Even though we controlled for the visual features when creating the scenes, the objects’ visual characteristics are not constant; this is an inevitable consequence of using realistic objects and scenes. Some triads also differ slightly in perspective or lighting. One measure to control for such low-level features in future studies would be the inclusion of a measure of visual salience (for example Itti et al., 1998; Garcia-Diaz et al., 2012) in order to control for possible effects of low-level features. Since the exact salience definition to be used may depend on the research question, and the development in the field of salience models is still ongoing (see Borji and Itti, 2013 for a review), we refrained from picking one particular measure to be included with the database. Object eccentricity and object size are additional relevant measures to include (Schomaker et al., 2017).

For the ratings of the entire scenes, we could not use the same set of scales as for the objects because interacting with an object (Interaction), owning an object (Desire to Own) and being familiar with an object (Recognizability) do not directly transfer to whole scenes. We therefore created two new scales: Interaction was intended to be replaced by Spending Time and Exploration. The PCA results show that the motivational scales indeed loaded on the same factor. Although the main aim of this database is to provide motivational ratings of everyday objects in the context of natural scenes, we include the scene ratings for completeness.

One limitation of MONS is that according to ideas of subjective value, there are strong individual differences in preferences and thus valuation of objects/products (Menger, 1950; Stigler, 1950; Von Mises and Greaves, 2007; Hare et al., 2009; Bartra et al., 2013). Subjective values thus also depend on personal likes and dislikes. Moreover, valuation and value-based decisions are state-dependent (e.g., depending on metabolic state: Symmonds et al., 2010; Levy et al., 2013). For example, the motivational value of a hotdog depends strongly on whether someone eats meat and what their calorie intake is for that day. Despite such individual preferences and state-dependent effects, we found that the scales were interpreted consistently and objects rated similarly by the raters. Another limitation of the database is that except for gender, age, and the level of education, the socioeconomic status and cultural characteristics of the participants are not known. Based on the methods of participant recruitment, most subjects can be expected to be from a European or general Western background (Henrich et al., 2010).

## Conclusion

Motivational Objects in Natural Scenes provides a controlled set of common objects and scenes varying in motivational value that will be available for research on the effects of motivation on brain and behavior. In contrast to existing databases, the objects are available both in isolation and presented in a natural scene setting, while maintaining control of visual features such as lighting and visual angle and providing object location in the scene. All objects, scenes, and rating data can be downloaded free of charge at doi: 10.5281/zenodo.883110 for use in non-commercial research projects.

## Author Contributions

Study design: BW, JS, WE, and ER. Stimulus creation and data acquisition: ER and JS. Data analysis: JS and WE. Writing: JS, BW, and WE.

## Conflict of Interest Statement

The authors declare that the research was conducted in the absence of any commercial or financial relationships that could be construed as a potential conflict of interest.

## Footnotes

1 http://www.gimp.org
